# Effects of *in vitro* copper sulphate supplementation on the ejaculated sperm characteristics in water buffaloes (*Bubalus bubalis*)

**Published:** 2013

**Authors:** Mehdi Tabassomi, Sayed Mortaza Alavi-Shoushtari

**Affiliations:** Department of Clinical Sciences, Faculty of Veterinary Medicine, Urmia University, Urmia, Iran.

**Keywords:** Buffalo, Copper sulphate, Semen extender

## Abstract

This study was carried out to investigate effects of copper sulphate (CuSO_4_) additive to semen extenders on sperm parameters: progressive motility, viability, membrane integrity and DNA damage, after semen dilution and cryopreservation. Semen samples of 5 buffalo bulls of 3-5 years old were collected at 5 different occasions during the autumn 2011. A total number of 25 samples were used in each examination. Sperm progressive motility and viability were measured at 0 (T_0_), 60 (T_1_) and 120 (T_2_) min after diluting semen in tris-citric acid extender containing 0 (control), 0.004, 0.008, 0.016, 0.032 and 0.064 mg L^-1^ CuSO_4_. Later, semen was diluted in a tris-citric acid-egg yolk-glycerol extender containing the same amounts of CuSO_4_, cooled to 4 ˚C and kept refrigerated for 4 hr to equilibrate, sperm progressive motility, viability, membrane integrity and DNA damage were estimated. Then, semen was packed in 0.5 mL French straws and frozen in liquid nitrogen. Later, the frozen semen was thawed in 37 ˚C water bath for 30 sec, and the same parameters as well as total antioxidant capacity (TAC) of the frozen-thawed semen were estimated. The results showed that copper additive at the rate of 0.032 mg L^-1^ gives a better protection of sperms through the process of dilution, equilibration and freeze-thawing than that in control and other Cu concentrations, while 0.064 mg L^-1^ CuSO_4_ had deleterious effect on the sperm.

## Introduction

Semen cryopreservation is the most important section of artificial insemination programs; it allows preservation of semen fertility for a long time. In this procedure, however, many sperm lose their motility and other parameters which lead to a low fertility rate. Many studies have demonstrated membrane lipid peroxidation (LPO) as one of the causes of defective sperm function in liquid semen preserved at 4 ˚C,^1^ and cryopreserved semen.^2^^-^^4^ Attempts have been made to preserve sperm parameters, particularly sperm motility, by adding some elements and materials to the semen before freezing.^5^^-^^7^

Copper is the metal cofactor for a variety of enzymes – amine oxidase, copper – dependent superoxide dismutase, cytochrome oxidase and tyrosinase. Copper is involved in dismutation, hydroxylation and oxygenation reactions. However, excess copper can oxidize proteins and lipids, bind to nucleic acids and enhance the production of free radicals and reduces the oxidative processes and glucose consumption, which reduces or abolishes sperm motility.^8^

Copper/zinc superoxide dismutase (Cu/Zn SOD) has been considered the primary antioxidant defense in cells. Three distinct types of SODs have been identified in mammals; two isoforms have copper (Cu) and zinc (Zn) in their catalytic center and are located either in the cytoplasm (Cu/Zn SOD) or in the extracellular elements. Reactive oxygen species (ROS) at minimum levels has been reported to have a variety of physiological roles such as in sperm capacitation and antimicrobial defense. Spermatozoa are highly sensitive to ROS-induced damage, while strong expression of Cu/Zn SOD has been observed in spermatogonia and they are tolerant to ROS. Zinc ion in Cu/Zn SOD is considered less critical and can be substituted with cadmium, mercury or cobalt ions, while no other metal can replace copper in restoring catalytic function. Among SOD isotypes, Cu/Zn SOD activity has been found to be higher in seminal plasma in mammals including buffaloes.^9^^,^^10^

Van Niekerk and Van Niekerk found that the testes development of rams suffering from a severe copper deficiency was slower than that in the control group. Histological examinations of the testes of the rams which suffered from a severe copper deficiency revealed that the seminiferous tubules were less developed and less active than those of the control group. This was mainly due to the inactivity of the Sertoli cells which contained only a small volume of cytoplasm in copper deficient rams.^11^ Copper appears to be involved in spermatozoa motility and it may also act at the pituitary receptors which control the release of LH. In the seminal fluid, the level of copper appears to fall in cases of azoospermia and to increase in oligo- and asthenozoospermia.^12^

This study was conducted to investigate the effects of *in vitro* copper supplementation on the progressive motility, viability, sperm membrane integrity and DNA stability of the spermatozoa in buffalo bulls’ semen during semen processing and freezing with the aim of finding a procedure to have an improved semen quality after freeze-thawing. 

## Materials and Methods

**Semen collection and processing. **Semen samples of five buffalo bulls of 3-5 years old kept in Buffalo Breeding Center of north-west of Iran (37˚ 33΄ N, 45˚ 4΄ E) were collected by a bovine pattern artificial vagina at five different occasions during the autumn 2011. A total number of 25 samples were used in each examination. Semen volume was recorded and sperm progressive motility and viability of fresh semen were measured at 0 (T_0_) (immediately after dilution), 60 (T_1_) and 120 (T_2_) min after diluting semen in tris-citric acid extender (tris 2.660 g, glucose 1.200 g, citric acid 1.390 g, cysteine 0.139 g, distilled water up to 100 mL) containing 0 (control), 0.004, 0.008, 0.016, 0.032 and 0.064 mg L^-1^ CuSO_4_ (CuSO_4_, 5H_2_O, Scharlau Chemie SA, Sentimental, Spain). At the same time, the semen was diluted in a tris-citric acid egg yolk-glycerol extender containing the same amounts of CuSO_4_, and left for 2 hr to cool to 4 ˚C and kept refrigerated for 4 hr to equilibrate. Then, semen parameters (progressive motility, viability, membrane integrity and DNA damage) were estimated and the semen was packed in 0.5 mL French straws and frozen in liquid nitrogen according to Sukhato *et al*. ^13^ Later, the frozen semen was thawed in 37 ˚C water bath for 30 sec, and the same parameters, as well as total antioxidant capacity (TAC) of the frozen-thawed semen, were estimated.

**Sperm functional assays. **The following assays were conducted on diluted fresh, cooled and frozen-thawed buffalo semen.

**Progressive motility and Sperm viability. **Sperm progressive motility was estimated using a computer assisted system of analysis (CASA) (Hoshmand Fannavar [HFT] CASA, Version 6, Amirkabir Medical Engineering Co., Tehran). Sperm viability was estimated by counting live and dead spermatozoa after staining samples by eosin-nigrosin staining method^14^ using an Olympus light microscope (Model BX41, Olympus Co., Tokyo, Japan).

**Plasma membrane integrity. **Hypo-osmotic swelling test (HOST) was used to examine membrane integrity of sperm before and after freezing according to a method applied by Jeyendran *et al.*^15^ Briefly, the solution of HOS contained sodium citrate 0.730 g and fructose 1.350 g, dissolved in 100 mL distilled water, osmotic pressure = 150 mOsmol kg^-1^. The assay was performed mixing 50 µL of semen sample to 500 µL of HOS solution and incubated at 37 ˚C for 40 min. After incubation, a drop of semen sample was examined and 200 spermatozoa were counted for their swelling characterized by coiled tail indicating intact plasma membrane.

**Total antioxidant capacity. **Total antioxidant capacity of the frozen-thawed semen was estimated using a commercial kit (Antioxidant Capacity Assay Kit, Cayman Chemical Co. Ann Arbor, MI, USA).

**Sperm DNA damage. **DNA damage was detected using Acridine Orange staining technique, according to the method of Katayose *et al.*^16^ First, spermatozoa were smeared on the glass slide. After being air dried, the samples were treated with acid alcohol (methyl alcohol-glacial acetic acid 3:1, v/v) for 2 hr. Immediately after air drying, approximately 1 mL of working solution containing 0.019% acridine orange (3, 6-bis [dimethylamino] acridine, hemi [zinc chloride] salt, Sigma Chemical Co., St. Louis, MO, USA.) was mounted on each slide glass for 5 min at room temperature and the samples were then washed with distilled water. The samples were observed under an epifluorescent microscope (Model GS7, Nikon Co., Tokyo, Japan) immediately after a coverslip was put in position. A total number of 100 to 200 spermatozoa were observed and classified by type as green (intact DNA) or red (damaged DNA) based on differences in their fluorescent color.

**Statistical analysis. **The data was analyzed using PASW software (Version 18 for windows, SPSS Inc., Chicago, IL, USA) computer program. Statistical mean and standard error of mean (SEM) were calculated for each group and compared with the others by One-way ANOVA and Duncan’s homogeneity tests were used for the comparison of the means, and the significance was set at *p* ≤ 0.05.

## Results

**Diluted fresh semen. **The results are summarized in Table 1. The sperm progressive motility and viability after adding 0.032 mg L^-1^ CuSO_4_ to the extender were preserved at T_1_ and T_2_ compared with controls and showed the highest values (progressive motility of 87.20 ± 0.90% vs. 84.80 ± 1.50% at T_0_ and 85.70 ± 1.00% vs. 77.40 ± 1.50% at T_2_; and viability of 88.40 ± 1.20% vs. 86.10 ± 1.20% at T_0_ and 86.30 ± 0.90% vs. 81.70 ± 1.10% at T_2_) (Table 1), that were not affected by the time lapse. However, by addition of 0.064 mg L^-1^ CuSO_4_, there was a significant decrease in these parameters compared with the controls (progressive motility of 75.20 ± 1.20% vs. 84.80 ± 1.50%; and viability of 77.80 ± 1.20% vs. 86.10 ± 1.20%) (Table 1) that continued by time lapse.

**Diluted equilibrated semen. **In diluted equilibrated semen the highest sperm progressive motility (80.40 ± 1.80%), viability (82.20 ± 1.20 %) and sperm membrane integrity (82.90 ± 0.70%) values were observed in 0.032 mg L^-1^ CuSO_4_ added extender (Table 2), but the number of normal DNA cells (green cells) in this dilution was not statistically different from other concentrations. Addition of 0.064 mg L^-1^ CuSO_4_ to the extender had an adverse effect (Table 2).

**Frozen-thawed semen. **In frozen-thawed semen, the highest sperm progressive motility (51.90 ± 1.90%), viability (65.70 ± 1.30%), sperm membrane integrity (62.90 ± 1.50%) values, which were statistically different from those of control, were observed in 0.032 mg L^-1^ CuSO_4_ added extender, and also, the least number of damaged DNA cells was obtained in this dilution (Table 3). Moreover, addition of 0.016 and 0.032 mg L^-1^ CuSO_4_ to the freezing extender, significantly (*p* < 0.05) increased the total antioxidant capacity of the frozen-thawed spermatozoa compared with that of the base extender (67.70 ± 1.30 and 69.30 ± 1.60 µmol L^-1^ vs. 63.30 ± 0.80 µmol L^-1^, respectively).

**Table 1:** Effects of different copper sulphate concentrations on the progressive motility of spermatozoa in fresh semen at different times (Mean ± SEM) (n = 25).

**Parameters**		**Copper sulphate concentration (mg L** ^-1^ **)**					
	**Time**	**0 (Control)**	**0.004**	**0.008**	**0.016**	**0.032**	**0.064**
**Motility (%)**	T_0_	84.80 ± 1.50 ^a^	84.20 ± 1.40 ^a^	85.00 ± 1.80 ^a^	85.40 ± 1.20 ^a^	87.20 ± 0.90 ^a^	75.20 ± 1.20 ^b^
	T_1_	80.80 ± 1.30 ^a^	81.00 ± 1.40 ^a^	83.50 ± 1.60 ^ab^	81.10 ± 1.30 ^a^	85.50 ± 1.10 ^b^	71.70 ± 1.20 ^c^
	T_2_	77.40 ± 1.50 ^a^	78.90 ± 1.20 ^ab^	78.10 ± 1.10 ^ab^	81.20 ± 0.40 ^b^	85.70 ± 1.00 ^c^	67.90 ± 1.20 ^d^
**Viability (%)**	T_0_	86.10 ± 1.20 ^a^	86.30 ± 1.20 ^a^	87.30 ± 1.50 ^a^	87.20 ± 1.00 ^a^	88.40 ± 1.20 ^a^	77.80 ± 1.20 ^b^
	T_1_	83.80 ± 1.50 ^a^	84.20 ± 1.20 ^a^	85.60 ± 1.40 ^a^	86.00 ± 1.00 ^a^	86.80 ± 1.10 ^a^	74.00 ± 1.30 ^b^
	T_2_	81.70 ± 1.10 ^ab^	80.20 ± 1.40 ^a^	83.50 ± 1.20 ^abc^	84.70 ± 1.30 ^bc^	86.30 ± 0.90 ^c^	71.70 ± 0.90 ^d^

Superscript letters (a, b and c) indicate significant differences (*p *< 0.05) within rows. T_0_: 0; T_1_:60 and T_2_:120 min after diluting semen.

**Table 2:** Effect of different copper sulphate concentrations on sperm parameters after equilibrium time (Mean ± SEM) (n = 25).

**Parameters**	**Copper sulphate concentration (mg L** ^-1^ **)**					
	**0 (Control)**	**0.004**	**0.008**	**0.016**	**0.032**	**0.064**
**Motility (%)**	71.40 ± 1.90 ^a^	73.20 ± 1.90 ^ab^	75.10 ± 1.90 ^abc^	77.30 ± 1.80 ^bc^	80.40 ± 1.80 ^c^	60.90 ± 1.90 ^d^
**Viability (%)**	78.00 ± 1.50 ^a^	78.50 ± 1.50 ^a^	79.20 ± 1.50 ^a^	80.60 ± 1.40 ^a^	82.20 ± 1.20 ^a^	72.70 ± 1.00 ^b^
**Membrane Integrity (%)**	79.00 ± 1.20 ^ab^	78.60 ± 1.20 ^ab^	80.90 ± 1.20 ^bc^	81.70 ± 1.20 ^bc^	82.90 ± 0.70 ^c^	76.10 ± 1.20 ^a^
**Damaged DNA (%)**	3.00 ± 0.25 ^a^	3.40 ± 0.21 ^a^	3.40 ± 0.20 ^a^	3.40 ± 0.24 ^a^	3.00 ± 0.25 ^a^	4.10 ± 0.21 ^b^

Superscript letters (a, b and c) indicate significant differences (*p* < 0.05) within rows.

**Table 3:** Effect of different copper sulphate concentrations on sperm parameters after thawing (Mean ± SEM) (n = 25).

**Parameters**	**Copper sulphate concentration (mg L** ^-1^ **)**					
	**0 (Control)**	**0.004**	**0.008**	**0.016**	**0.032**	**0.064**
**Motility (%)**	40.50 ± 1.70 ^a^	41.60 ± 1.70 ^a^	43.50 ± 1.70 ^ab^	48.00 ± 1.70 ^bc^	51.90 ± 1.90 ^c^	35.00 ± 1.60 ^d^
**Viability (%)**	60.10 ± 1.50 ^a^	60.60 ± 1.50 ^a^	61.70 ± 1.40 ^ab^	62.90 ± 1.40 ^ab^	65.70 ± 1.30 ^b^	54.80 ± 1.60 ^c^
**Membrane Integrity (%)**	56.60 ± 1.70 ^a^	58.50 ± 1.70 ^ab^	59.40 ± 1.70 ^ab^	61.30 ± 1.70 ^ab^	62.90 ± 1.50 ^b^	51.50 ± 1.80 ^c^
**Damaged DNA (%)**	11.80 ± 0.31 ^a^	11.60 ± 0.43 ^a^	11.50 ± 0.42 ^a^	10.30 ± 0.49 ^b^	10.10 ± 0.47 ^b^	13.30 ± 0.26^c^
**TAC (µmol L** ^-1^ **)**	63.30 ± 0.80 ^a^	63.70 ± 1.00 ^a^	63.70 ± 1.00 ^a^	67.70 ± 1.30 ^b^	69.30 ± 1.60 ^bc^	57.90 ± 1.00 ^d^

Superscript letters (a, b and c) indicate significant differences (*p* < 0.05) within rows.

## Discussion

Sperm processing and cryopreservation involved in the semen preservation and artificial insemination reduces sperm motility and fertility^17^^-^^19^ and also decreases the antioxidant defense capacity of semen.^20^^-^^23^ The antioxidant capacity in sperm cells, however, is insufficient in preventing lipid peroxidation during the freeze-thawing process. Thus, antioxidants present in the seminal plasma are the most important form of protection available to spermatozoa against ROS.^24^^-^^26^ Antioxidant supplementation of semen extender during liquid storage or cryopreservation of the bull and other mammalian spermatozoa and its beneficial effects has been reported.^5^^,^^27^^,^^28^ Copper as an antioxidant, particularly as a cofactor of some enzymes, such as Cu/Zn SOD, plays a major role in the protection of spermatozoa against peroxidative damage of its cellular enzymes and structures from reactive oxygen species.^25^^,^^29^^,^^30^ The effect of copper content of seminal plasma on sperm parameters including motility and viability in buffaloes has been reported;^10^ but there is little information available about its *in vitro* effects. 

This study was designed to investigate the effects of different amounts of copper supplementation of the extenders and examine its *in vitro* effects in an attempt to find a way for preserving the quality of the sperm after freezing. Sperm progressive motility and viability, as most important parameters of sperm fertility, were evaluated immediately after semen dilution in different amounts of CuSO_4_ at different times to see the dose and time effects. Other parameters such as membrane integration, DNA damage and TAC were added to investigate equilibration and freeze-thawing effects.

The present study revealed that addition of CuSO_4_ to semen extender improved total antioxidant capacity, in a dose dependent manner. Copper therapy-associated improvement in sperm parameters included an increase in the seminal antioxidant capacity and reduction of oxidative stress status. Furthermore, it has been shown that Cu modulated the Cu/Zn SOD activity, and addition of Cu to the testicular cell culture increased the enzymatic activity of Cu/Zn SOD.^9^

Addition of 0.032 mg L^-1^ CuSO_4_ to the semen extender in the present study led to a significant increase in sperm progressive motility after equilibration; while progressive motility, viability and TAC of the semen were increased after freeze-thawing, number of sperms with damaged DNA were lowered at this stage. 

 A significant correlation between copper concentrations in human semen and sperm progressive motility and normal morphology has been reported.^31^ There are reports indicating that copper ion is essential for maximal superoxide dismutase activity which is considered as the principal antioxidant enzyme that may lead to less free radical formation during the spermatogenic process and to increased normal sperm population.^8^ However, low concentrations of ionic copper has been reported to cause a less marked fall in spermatozoa motility.^32^ Yuyan *et al*. reported on differences among progressive motility of the five experimental copper supplemented groups, but multiple logistic analyses did not show that higher or lower serum copper levels had a significant effect on human sperm quality.^33^

Machal *et al*. reported that positive correlation was found between the Cu concentration in the bovine blood plasma and the total number of sperm in ejaculate, the total number of sperm with progressive motility and between the Cu concentration in seminal plasma and sperm progressive motility in the ejaculate.^34^

There was a significant positive correlation between copper and zinc concentrations of seminal plasma and progressive motility percent of spermatozoa. Therefore, with increasing copper and zinc concentrations in rooster seminal plasma, the progressive motility percent of spermatozoa was also increased.^35^

Reportedly, after almost a year on copper deficient rations, rams produced ejaculates of lower volume, lower sperm concentration, poorer sperm motility and morphology than rams in the control group. After the copper deficiency was reversed, the above parameters reverted to normal. It was concluded that an experimentally induced copper deficiency produced reversible impairment of testicular function in rams.^36^

The study of Wong *et al*. demonstrated a weak but significant positive correlation between blood Cu concentrations and sperm motility.^37^ Ackerman *et al.*, however, demonstrated an adverse effect of high concentrations of Cu on sperm morphology in impala living in the Kruger National Park, South Africa.^38^ Copper concentrations determined in Wong’s study were within the normal physiologic range and it is known that Cu is an essential trace element that plays an important role in several enzymes such as superoxide dismutase.^37^

These results showed that copper supplementation of semen extenders seemed to be a promising procedure for preserving spermatozoa quality during cryo-preservation processes. 

It was shown that adding 0.032 mg L^-1^ CuSO_4_ to the extender gave a better sperm preservation and although a bigger sample population was needed for a definite statement, it could be concluded that copper supplementation of semen extenders might help preserve the sperm quality, progressive motility, viability, membrane integrity and total antioxidant capacity upon freezing processes and led to a lesser degree of sperm with damaged DNA after semen freeze-thawing, which in turn, resulted in a higher semen fertility. However, addition of higher copper concentrations (0.064 mg L^-1^) was detrimental to spermatozoa.
